# *AMFR* and *DCTN2* genes cause transplantation resistance of adipose-derived mesenchymal stem cells in type 1 diabetes mellitus

**DOI:** 10.3389/fphar.2022.1005293

**Published:** 2022-10-04

**Authors:** Michiko Horiguchi, Yuya Tsurudome, Kentaro Ushijima

**Affiliations:** Division of Pharmaceutics, Faculty of Pharmaceutical Sciences, Sanyo-Onoda City University, Yamaguchi, Japan

**Keywords:** stem cell, adipose-derived mesenchymal stem cells (ADSCs), type 1 diabetes, *AMFR*, *DCTN2*

## Abstract

Type 1 diabetes mellitus (T1DM) is characterized by pancreatic beta cell destruction by autoantibodies and other factors, resulting in insulin secretion deficiency. Therefore, beta cell regeneration would be necessary to cure the disease. Nevertheless, the impact of type 1 diabetes on the stemness and transplantation efficiency of stem cells has not been previously described. In this study, we used next-generation sequencing to identify genes differentially expressed in T1DM adipose-derived stem cells (T1DM ADSCs) that originate from patients with type 1 diabetes. Furthermore, we evaluated their effects on transplantation efficiency following xenotransplantation into immunodeficient mice. In the T1DM ADSCs transplant group, the volume and weight of the graft were significantly reduced and the transplant efficiency was reduced. Next-generation sequencing and quantitative PCR results showed that T1DM ADSCs had significantly increased expression of *AMFR* and *DCTN2*. *AMFR* and *DCTN2* gene knockdown in T1DM ADSC significantly restored cell proliferation and stem cell marker expression. Therefore, transplantation of T1DM ADSCs, in which *AMFR* and *DCTN2* were knocked down, into immunodeficient mice improved transplant efficiency. This study revealed that *AMFR* and *DCTN2* can reduce transplantation efficiency of T1DM ADSCs. Focusing on *AMFR* and *DCTN2* is expected to increase the efficiency of stem cell transplantation therapy for diabetic patients.

## Introduction

Type 1 diabetes mellitus (T1DM) is a disease characterized by pancreatic beta cell destruction by autoantibodies and other factors, resulting in insulin secretion deficiency (Nathan et al., 1993; Foster et al., 2019). For patients with T1DM, insulin replacement therapy is essential, and regenerative therapy to replace or repair damaged beta cells would be useful in disease treatment (Speight et al., 2010; Foster et al., 2018). Currently, the application of stem cells or beta cells differentiated from stem cells is being investigated for regenerative therapy of T1DM (Brusko et al., 2021). Stem cell transplantation sources in the literature include mesenchymal stem cells, embryonic stem cells (ESCs) and induced pluripotent stem cells (iPSCs) (Kondo et al., 2018; Bao et al., 2021; Hogrebe et al., 2021).

The present study focuses on adipose-derived mesenchymal stem cells (ADSCs), which have been reported to differentiate into various cells, including chondrocytes, adipocytes, myogenic cells, and endothelial cells (Huang et al., 2021). Compared to bone marrow-derived mesenchymal stem cells (BMSCs), ADSCs are more abundant in the tissue and provide a useful resource for regenerative therapy since ADSCs secrete high levels of vascular endothelial growth factor (Mareschi et al., 2006). Therefore, ADSCs have the advantage of being available in large quantities from multiple tissues and facilitating the construction of cell banks.

We previously described that T1DM ADSCs show distinct cellular degeneration compared with stem cells derived from type 2 diabetes mellitus (T2DM), such as considerable spherical endoplasmic reticulum hypertrophy (Horiguchi et al., 2021a; Horiguchi et al., 2021b; Horiguchi et al., 2021c). In addition, our group has previously reported that exosomes from T1DM ADSCs are larger but fewer, and have a higher tetraspanin CD9-positive ratio (Horiguchi et al., 2021a). Nevertheless, the genes differentially expressed in T1DM ADSCs have not been identified, and the effects of T1DM on their stemness and transplantation efficiency have not been elucidated.

In the current study, we aimed to identify genes differentially expressed in T1DM ADSCs and to assess their effects on cell stemness and transplantation efficiency. The importance of this study is to demonstrate the transplantation resistance of T1DM ADSCs *in vivo* and to identify specific target genes to improve transplantation resistance.

We hypothesized that stem cells derived from patients with type 1 diabetes may have lower transplantation efficiency than stem cells derived from healthy individuals. The research question of this study is to identify the genes that determine the transplantation efficiency of stem cells derived from patients with type 1 diabetes.

## Materials and methods

### Materials

The materials were shown in the Supplementary Table S1.

### Adipose-derived stem cell culture

Primary ADSCs were purchased from Lonza [Adipose-Derived Stem Cells (Lonza, SC, United States)]. The source of ADSCs was a healthy volunteer (Lot number: 19TL261894, Female, 66 years old) and a patient with type 1 diabetes mellitus (Lot number: 1F4104, Female, 62 years old).

Normal ADSCs and T1DM ADSCs were seeded at a density of 2000–6000 cells/cm^2^. The ADSCs were cultured in Adipose-Derived Stem Cells Growth Medium BulletKit™ (Lonza, SC, United States) in an incubator with 5% CO_2_ and at 37°C. ADSCs were positive for CD13, CD29, CD44, CD73, CD90, CD105, and CD166, which confirmed them as mesenchymal stem cells. ADSCs up to the 5th passage expressed stem cell markers and were used for experiments.

### Evaluation of stem cell transplantation efficiency in immunodeficient mice

Normal ADSC and T1DM ADSC were cultured in the form of spheroids using Matrigel for spheroid culture [Corning Matrigel Basement Membrane Matrix For organoid formation Phenol red free (Corning, NY, United States)]. Stem cell spheroids were mixed with Matrigel for transplantation in a 1:1 ratio. 300 μl of a transplantation mixture containing 10^7^ cells of ADSCs was transplanted subcutaneously into the back of immunodeficient mice (BALB/cAJcl-nu/nu). 2 weeks after the ADSC transplantation, the width, depth, and height of the transplant sites were measured with a caliper, and the volume (mm^3^) was calculated. The weight (mg) of the graft removed 3 weeks after ADSC transplantation was weighed with a precision electronic balance.

### Expression variation gene analysis method by next-generation sequence

Total RNA was extracted from Normal ADSC and T1DM ADSC using the RNeasy Mini Kit (QIAGEN, Venlo, Netherlands). Using 100 ng of Total RNA, poly (A) RNA was extracted and fragmented using NEBNext Poly (A) mRNA Magnetic Isolation Module and NEBNext Ultra II RNA Library Prep Kit for Illumina. A reverse transcription reaction was performed on the fragmented poly (A) RNA using the NEBNext First Strand Synthesis Enzyme Mix of the NEBNext Ultra II RNA Library Prep Kit (Illumina) to prepare cDNA, and the NEBNext Adapter was subsequently added. The prepared cDNA was amplified by PCR to prepare a library. The cDNA region 75 bp and the barcode sequence were analyzed with Illumina Next Seq using fragment analysis. The Database for Annotation, Visualization, and Integrated Discovery (DAVID) was used for pathway analysis of expression-variable genes.

### Quantitative PCR method

mRNA was extracted from Normal ADSC and T1DM ADSC using the RNeasy Mini Kit. The extracted mRNA was reversed transcribed using the PrimeScript RT reagent Kit to prepare a template cDNA. SYBR Premix Ex Taq™ II was used for quantitative PCR. The primer used was purchased from TAKARA Perfect Real Time Primer support system. The cDNA, SYBR Premix Ex Taq™ II, Forward and Reverse primers were mixed, and a PCR reaction was performed using the quantitative PCR device StepOne-Plus-01. The reaction was carried out at 95°C for 30 s, and a cycle of 95°C for 5 s and 60°C for 60 s was repeated 40 times. The expression level of the target gene was calculated using the calibration curve, and the expression ratio was calculated using GAPDH as an internal standard. We use the GAPDH as a housekeeping gene. The gene expression rates were shown the fold over the gene expression of GAPDH. The list of primer was shown in the Supplementary Table S2.

### *AMFR* and *DCTN2* gene knockdown

The *AMFR* and *DCTN2* genes were knocked down using siRNA. For siRNA, Silencer^®^ Select Pre-Designed siRNA (ambion by life technologies) was used. The list of siRNA was shown in the Supplementary Table S3. The siRNA was diluted to the recommended concentration in serum-free medium and mixed with the transfection reagent Lipofectamine™ LTX Reagent and Plus™ Rreagent (Invitrogen CA, United States). The mixed solution was added to the cultured ADSC every 48 h and cultured in a CO_2_ incubator. Knockdown was confirmed by quantitative PCR in which the target gene expression was reduced by 80% or more.

### Evaluation of cell viability

ADSC cell viability were determined by BOSTER’s MTT cell proliferation and cytotoxicity assay kit (BOSTER biological technology, CA, United States). For the MTT assay, 4,000 cells were seeded in each well of a 96-well plate, cultured in a label reaction solution for 4 h, and the absorbance of the formazan dye was measured at 570 nm.

### Evaluation of stemness by flow cytometry

ADSC was adjusted to 1 × 10^6^ cells/ml and blocked using Fc receptor blocking reagents. 10 μl each of PerCP-labeled CD105 and APC-labeled CD90 antibodies and 10 μl of PE-labeled Negative Marker Cocktail (including CD45, CD34, CD11b, CD79A, and HLA-DR antibodies) were incubated with the cells at room temperature for 45 min. The cells were washed and the cell pellet was resuspended in 400 μl Staining Buffer. The sample was analyzed with a flow cytometer SA3800. The acquired analysis data was analyzed by FLOWJO to calculate the percentage of positive cells.

### Statistical analysis

The values are expressed as mean ± SD. Significant differences between two groups were evaluated using the two-tailed, unpaired *t*-test with Welch’s correction. Statistical significance in Figure 3 was determined by Two-way ANOVA. Statistical analyses were carried out using Prism9 software. The *p*-values are indicated in each figure legend. *p* < 0.05 was considered to indicate statistical significance.

## Results

### Decreased transplantation efficiency of type 1 diabetes mellitus adipose-derived stem cells

**FIGURE 1:**
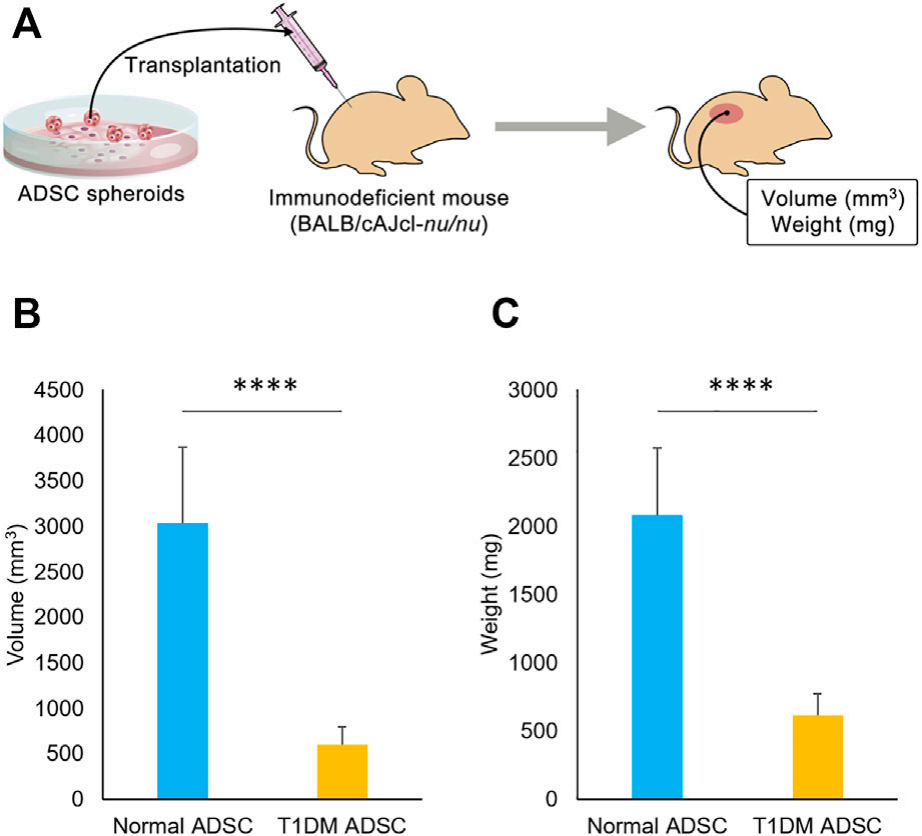
Transplantation outcome in immunodeficient mice. **(A)** Diagram showing transplantation of ADSCs spheroids to immunodeficient mice. **(B)** Graft volume (mm^3^) 2 weeks after stem cell transplantation. **(C)** Graft weight (mg) 3 weeks after stem cell transplantation. Three independent experiments were performed for the assay. **(B)** The *p*-value was <0.0001.**(C)** The *p*-value was <0.0001. Normal ADSCs and T1DM ADSCs were seeded at a density of 6000 cells/cm^2^.

To assess the difference in transplantation efficiency, normal ADSCs and T1DM ADSCs were mixed with matrigel and growth factors and transplanted into the dorsal subcutaneous region of BALB/cAJcl-nu/nu immunodeficient mice (Figure 1A). 2 weeks after the transplantation, the volume of the graft was 601 ± 200 mm^3^ in the T1DM ADSC transplantation group, which was approximately five times lower than that of the normal ADSC transplantation group (3,036 ± 837 mm^3^) (Figure 1B). In addition, the graft weight 3 weeks after the transplantation was 615 ± 160 mg in the T1DM ADSC group, which was approximately three times lower than that of the normal ADSC group (2081 ± 493 mg) (Figure 1C). In summary, T1DM ADSCs have significantly lower graft volume and weight and lower graft efficiency compared to normal ADSCs.

### Modified expression genes in type 1 diabetes mellitus adipose-derived stem cells

**FIGURE 2:**
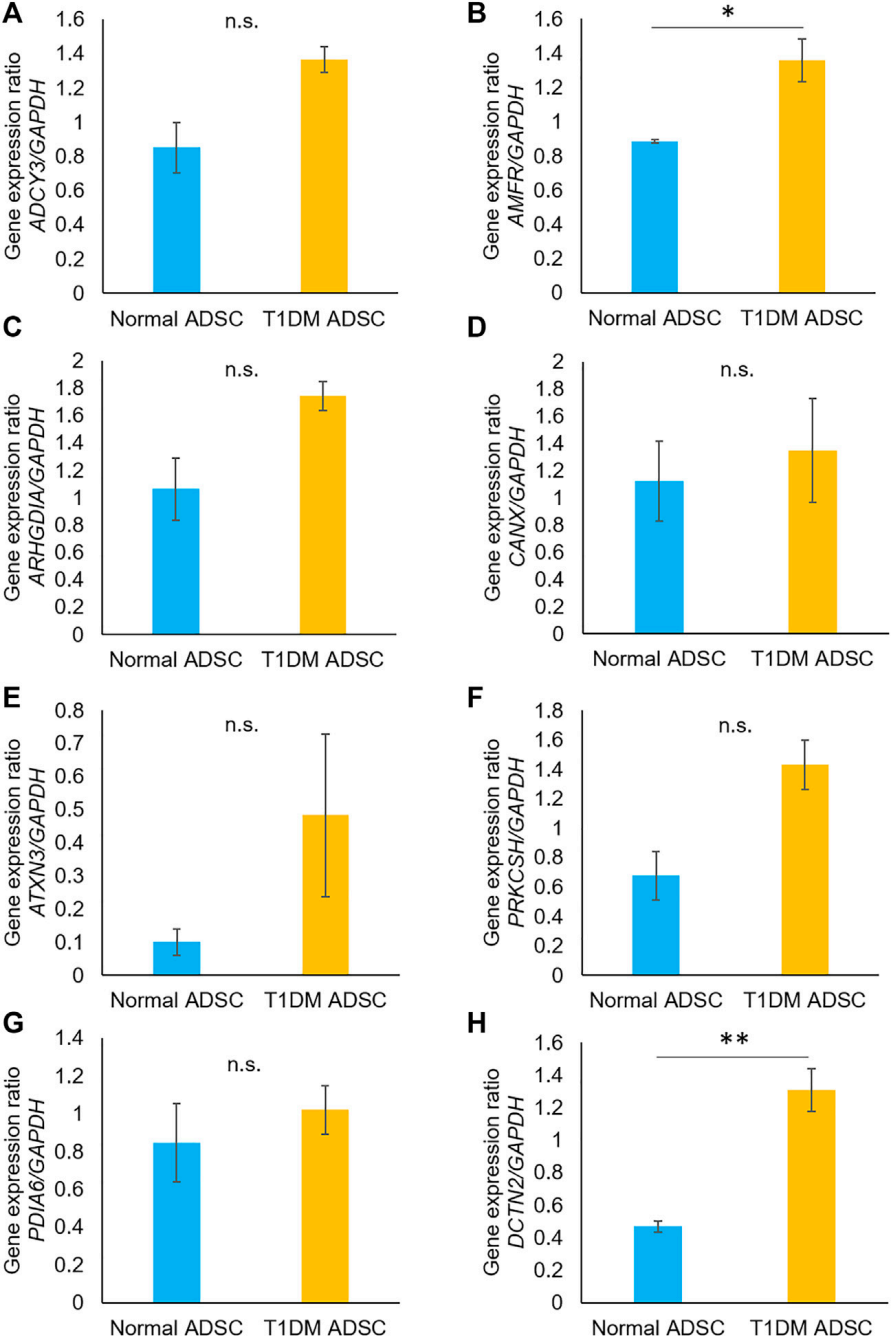
Gene expression analysis using quantitative PCR on human adipose tissue-derived mesenchymal stem cells. The gene expression of the eight genes identified in next-generation sequencing was evaluated by quantitative PCR. The gene variation graphs were marked for each gene **(A)** *ADCY3*, **(B)** *AMFR*, **(C)** *ARHGDIA*, **(D)** *CANX*, **(E)** *ATXN3*, **(F)** *PRKCSH*, **(G)** *PDIA6*, **(H)** *DCTN2*. Gene expression was expressed as a ratio to the housekeeping gene GAPDH. The light blue bars present values for normal ADSC, and the orange bars show values for T1DM ADSC. Three independent experiments were performed for the assay. Welch’s *t*-test was used for statistical analysis. The *p*-value of *AMFR* was 0.0363, and the *p*-value of *DCTN2* was 0.0071. Normal ADSCs and T1DM ADSCs were seeded at a density of 5000 cells/cm^2^.

In order to identify the genes in T1DM ADSCs, a comprehensive gene analysis was performed using next-generation sequencing. The analysis extracted a total of 185 genes showing more than 20-fold difference in expression in T1DM ADSCs compared to normal ADSCs. The Kyoto Encyclopedia of Genes and Genomes (KEGG) pathway analysis for these 185 extracted genes was then performed using the Database for Annotation, Visualization, and Integrated Discovery (DAVID). The analysis revealed the involvement of the vasopressin-regulated water reabsorption pathway and the protein processing in the endoplasmic reticulum (ER) pathway, which are key signaling pathways for vesicle formation (Table 1). In addition, the expression of three genes related to the vasopressin-regulated water reabsorption pathway, *ADCY3*, *DCTN2*, and *ARHGDIA*, and five genes related to the protein processing in the ER pathway, *ATXN3*, *AMFR*, *CANX*, *PDIA6*, and *PRKCSH*, were found to be changed (Table 2). The gene expression of these eight genes were then examined by quantitative PCR. The results showed that *AMFR* and *DCTN2* gene expression were significantly increased in T1DM ADSCs compared to normal ADSCs (Figure 2).

**TABLE 1:** KEGG pathway analysis results for genes with variable expression using next-generation sequencing.

Term of KEGG _pathway	Count	%	*p*-value	Benjamini
Vasopressin-regulated water reabsorption	3	1.6	0.072	1
Protein processing in endoplasmic reticulum	5	2.7	0.089	1

**TABLE 2:** Genes with variable expression in the vasopressin-regulated water reabsorption pathway and in protein processing in the endoplasmic reticulum pathway.

Name	Gene ID	Expression value	T1DM ADSC expression value	Ratio (normal/T1DM)
ADCY3	ENSG0000128031	4.08	0.13	30.42
*AMFR*	ENSG0000159461	12.94	0.10	133.84
ARHGDIA	ENSG0000141522	31.91	1.52	21.04
CANX	ENSG0000127022	34.35	1.44	23.88
ATXN3	ENSG0000066427	0.12	2.56	0.05
PRKCSH	ENSG0000130175	1.02	20.80	0.05
PDIA6	ENSG0000143870	17.37	0.59	29.29
*DCTN2*	ENSG0000175203	21.65	0.78	27.58

### Effects of *AMFR* and *DCTN2* genes on cell viability and stem cell characteristics of type 1 diabetes mellitus adipose-derived stem cells

**FIGURE 3:**
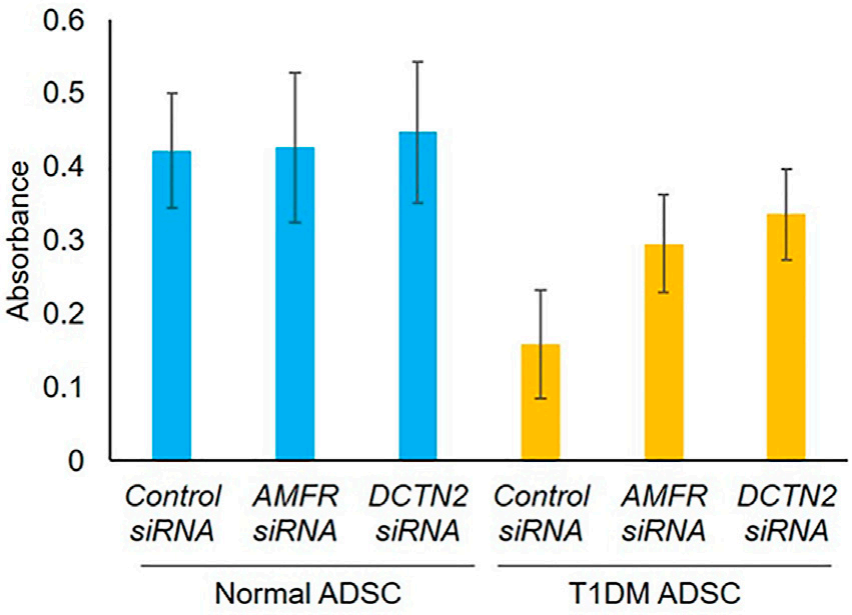
Evaluation of cell viability on human adipose tissue-derived mesenchymal stem cells. Effect of *AMFR* and *DCTN2* gene knockdown on cell viability by the MTT assay method. Three independent experiments were performed for the assay. Normal ADSCs and T1DM ADSCs were seeded at a density of 2000 cells/cm^2^.

First, the effect of *AMFR* and *DCTN2* on ADSC viability was assessed. Viability of T1DM ADSCs was significantly lower than that of Normal ADSC, but *AMFR* and *DCTN2* knockdown significantly improved the cell viability (Figure 3).

**FIGURE 4:**
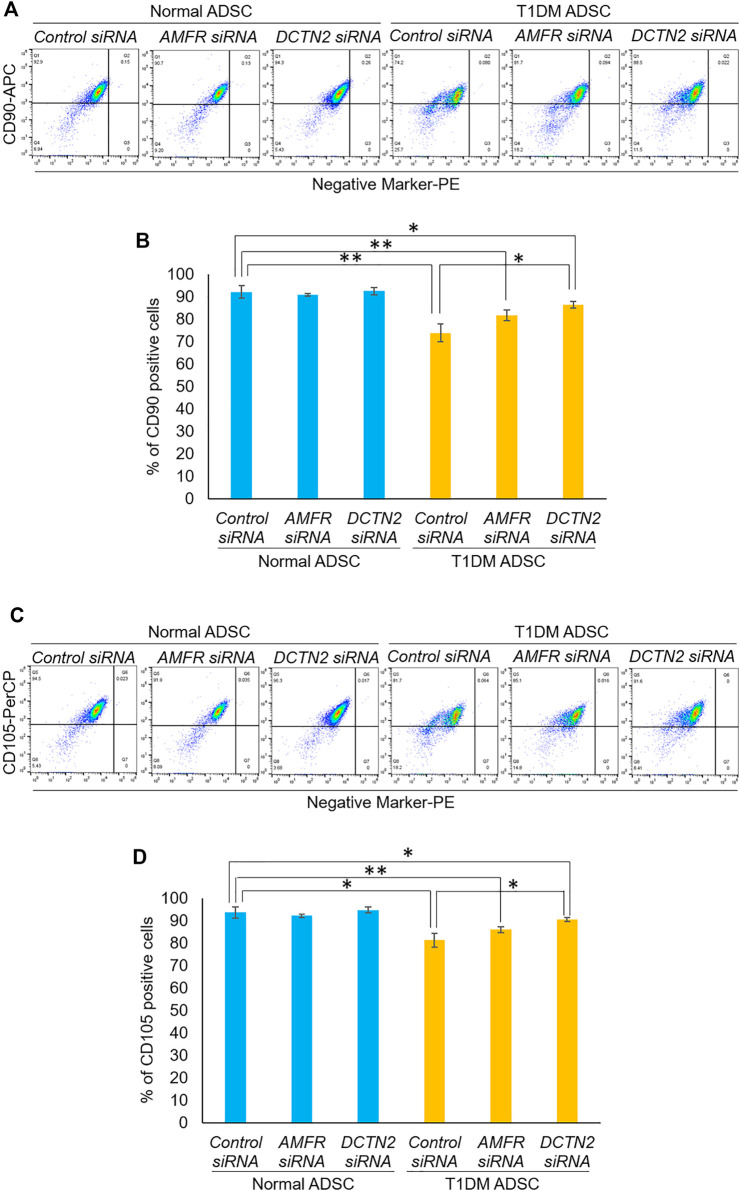
Evaluation of stemness on human adipose tissue-derived mesenchymal stem cells. Effect of *AMFR* and *DCTN2* gene knockdown on the positive rate of stem cell markers by flow cytometry. **(A)** Distribution of CD90, a marker of ADSCs in normal ADSCs and T1DM ADSCs. **(B)** Quantitative analysis of the percentage of CD90 positive cells. The *p*-values were 0.5779 (Normal ADSCs Control siRNA vs. Normal ADSCs *AMFR* siRNA), 0.8471 (Normal ADSCs Control siRNA vs. Normal ADSCs *DCTN2* siRNA), 0.0692 (T1DM ADSCs Control siRNA vs. T1DM ADSCs *AMFR* siRNA), 0.013 (T1DM ADSCs Control siRNA vs. T1DM ADSCs *DCTN2* siRNA), 0.0059 (Normal ADSCs Control siRNA vs. T1DM ADSCs Control siRNA), 0.0062 (Normal ADSCs *AMFR* siRNA vs. T1DM ADSCs *AMFR* siRNA), 0.0165 (Normal ADSCs *DCTN2* siRNA vs. T1DM ADSCs *DCTN2* siRNA). **(C)** Distribution of CD105, a marker of ADSCs in normal ADSCs and T1DM ADSCs. **(D)** Quantitative analysis of the percentage of CD105 positive cells. Data are presented as mean ± SD. Three independent experiments were performed for the assay. The *p*-values were 0.4997 (Normal ADSCs Control siRNA vs. Normal ADSCs *AMFR* siRNA), 0.608 (Normal ADSCs Control siRNA vs. Normal ADSCs *DCTN2* siRNA), 0.1182 (T1DM ADSCs Control siRNA vs. T1DM ADSCs *AMFR* siRNA), 0.0156 (T1DM ADSCs Control siRNA vs. T1DM ADSCs *DCTN2* siRNA), 0.0123 (Normal ADSCs Control siRNA vs. T1DM ADSCs Control siRNA), 0.0036 (Normal ADSCs *AMFR* siRNA vs. T1DM ADSCs *AMFR* siRNA), 0.0156 (Normal ADSCs *DCTN2* siRNA vs. T1DM ADSCs *DCTN2* siRNA). Normal ADSCs and T1DM ADSCs were seeded at a density of 5000 cells/cm^2^.

Next, we evaluated the effect of *AMFR* and *DCTN2* on stem cell marker expression. In the Control siRNA-treated group, T1DM ADSCs had significantly lower expression of the stem cell markers CD105 and CD90 compared to Normal ADSCs (Figure 4). This decrease in stem cell marker expression was significantly increased in the *AMFR* or *DCTN*2 siRNA-treated group compared to the Control siRNA-treated group (Figure 4). Based on the results above, *AMFR* and *DCTN2* cause a decrease in the ADSC proliferation stem cell marker expression.

### *AMFR* and *DCTN2* gene effects on transplant efficiency of type 1 diabetes mellitus adipose-derived stem cells

**FIGURE 5:**
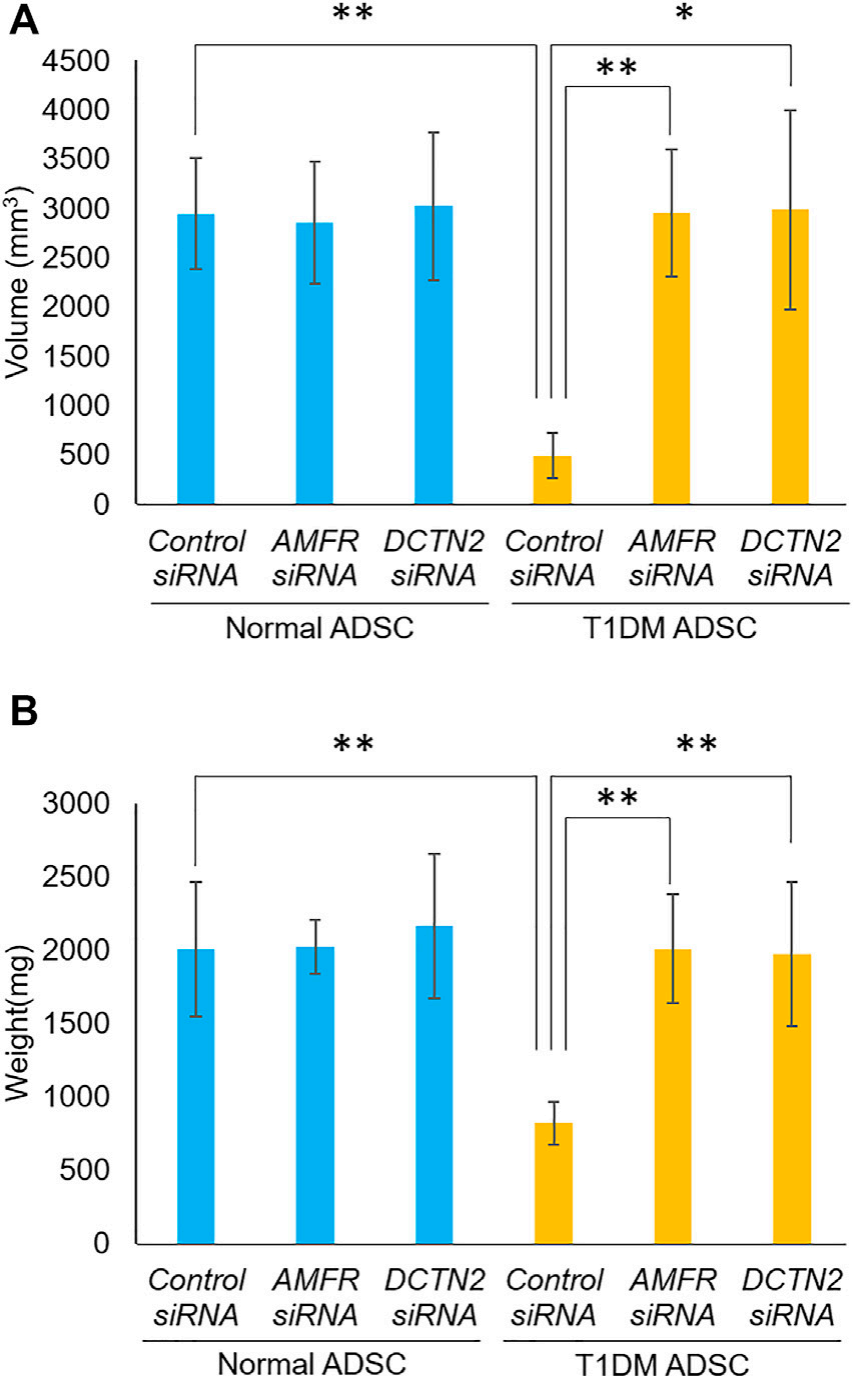
Transplantation outcome in immunodeficient mice. Effects of *AMFR* and *DCTN2* on stem cell transplantation efficiency. **(A)** Graft volume (mm^3^) 2 weeks after stem cell transplantation. **(B)** Graft weight (mg) 3 weeks after stem cell transplantation. **(A)** The *p*-value were 0.8883 (Normal ADSCs Control siRNA vs. Normal ADSCs *AMFR* siRNA), 0.9075 (Normal ADSCs Control siRNA vs. Normal ADSCs *DCTN2* siRNA), 0.0072 (T1DM ADSCs Control siRNA vs. T1DM ADSCs *AMFR* siRNA), 0.0273 (T1DM ADSCs Control siRNA vs. T1DM ADSCs *DCTN2* siRNA), 0.0046 (Normal ADSCs Control siRNA vs. T1DM ADSCs Control siRNA), 0.888 (Normal ADSCs *AMFR* siRNA vs. T1DM ADSCs *AMFR* siRNA), 0.9663(Normal ADSCs *DCTN2* siRNA vs. T1DM ADSCs *DCTN2* siRNA). **(B)** The *p*-value were 0.9641 (Normal ADSCs Control siRNA vs. Normal ADSCs *AMFR* siRNA), 0.7603 (Normal ADSCs Control siRNA vs. Normal ADSCs *DCTN2* siRNA), 0.0021 (T1DM ADSCs Control siRNA vs. T1DM ADSCs *AMFR* siRNA), 0.0081 (T1DM ADSCs Control siRNA vs. T1DM ADSCs *DCTN2* siRNA), 0.009 (Normal ADSCs Control siRNA vs. T1DM ADSCs Control siRNA), 0.9542 (Normal ADSCs *AMFR* siRNA vs. T1DM ADSCs *AMFR* siRNA), 0.6876(Normal ADSCs *DCTN2* siRNA vs. T1DM ADSCs *DCTN2* siRNA). Normal ADSCs and T1DM ADSCs were seeded at a density of 6000 cells/cm^2^.

Furthermore, the effects of *AMFR* and *DCTN2* on transplantation efficiency was evaluated. In the T1DM ADSC group treated with Control siRNA, the volume and weight of the transplant site were significantly reduced compared to the Normal ADSC group treated with Control siRNA (Figure 5). The *AMFR* and *DCTN2* siRNA-treated groups significantly increased the volume and weight of the transplant site (Figure 5). Based on the results above, *AMFR* and *DCTN2* cause decreased transplantation efficiency.

## Discussion

Over the past decade, controlled clinical trials have been conducted to assess the efficacy of stem cell therapy for T1DM (Cai et al., 2016). MSCs and ADSCs have been demonstrated to be able to ameliorate diabetes in animal models of T1DM (Chen et al., 2020). It was reported that MSCs and ADSCs treatment can preserve β-cell function in T1DM patients (Carlsson et al., 2015). However, it has not been clarified whether stem cells from patients with type 1 diabetes are suitable for transplantation. In the current study, the genes that are differentially expressed in T1DM ADSCs were identified, and their effects on stemness were evaluated. We found increased expression of *AMFR* and *DCTN2*, genes involved in vesicle formation. Furthermore, we showed that *AMFR* and *DCTN2* regulated the stemness and transplantation efficiency in T1DM ADSCs. The present study characterized stem cells derived from type 1 diabetes subjects and showed a difference in efficacy in stem cell transplantation.

Previous studies have not elucidated the genes that affect transplantation resistance of type 1 diabetes-derived stem cells. In this study, we used Next-generation sequencing analysis of T1DM ADSCs, and identified involvement of the vasopressin-regulated water reabsorption pathway and the protein processing in the ER pathway based on the KEGG (Kanehisa et al., 2017) pathways maps. Vasopressin is a hormone that is synthesized in the hypothalamus and secreted from the posterior pituitary gland (Buijs et al., 1983). Vasopressin interferes with diuresis by increasing the reabsorption of water in renal tubules (Koshimizu et al., 2012). The vasopressin-regulated water reabsorption pathway is a signaling pathway that regulates water reabsorption through endocytosis and exocytosis functions by aquaporins while it also contributes to the vesicle formation process (van Balkom et al., 2002). The protein processing in the ER pathway is an important signaling pathway for protein folding in the ER and regulating ER stress and apoptotic cell death (Hetz, 2012). Among the genes associated with these two pathways, *AMFR* and *DCTN2* showed significant differences based on quantitative PCR analysis. *AMFR* is a gene encoding a receptor associated with autocrine signaling, which is essential for overall cell signaling (Nakajima and Raz, 2020). *DCTN2* is a microtubule-associated gene, and the gene product can affect intracellular vesicle migration (Shen et al., 2018). Elucidation of the molecular mechanism by which *AMDR* and *DCTN2* regulate stem cells is still needed.

There was a decrease in transplantation efficiency of T1DM ADSCs. This could be explained by exosome degeneration and a decrease in cell viability and stemness, as suggested in the current study. Stem cells are characterized by their ability to self-renew and differentiate into different cell types (Simons and Clevers, 2011). In T1DM ADSCs, the proliferative potential and stemness of the cells were reduced compared to normal ADSCs, which may affect their transplantation efficiency. We also reported reduced transplantation efficiency in immunodeficient mice with adipose-derived stem cells from type 2 diabetic patients (T2DM ADSCs); (Horiguchi et al., 2021b) however, exosome degeneration was not observed in T2DM ADSCs. This may suggest that the mechanism of reduced transplantation efficiency in T1DM ADSCs may be different than in type 2 diabetes.

There were several limitations associated with the current study. First, as the stem cell transplantation effect was evaluated in immunodeficient mice that lack the immune components, a correlation with the transplantation efficiency in humans cannot be made. Although immunodeficient mice have been frequently used as xenotransplantation models, there are significant genetic and protein differences between mice and humans. Thus, verification of the transplantation efficiency of T1DM ADSCs in humans remains. Second, whether exosome degeneration occurs in ADSCs derived from all type 1 diabetic patients has not been verified. We selected normal ADSC from one subject and T1DM ADSC from another subject with a common background out of eleven donors in the present study. Finally, we have not clarified whether exosome degeneration and transplantation efficiency correlates with disease severity, duration, and age. Therefore, more donors are needed in future studies to evaluate the relationship between enlarged exosomes and disease characteristics.

This study characterized adipose tissue stem cells derived from a type 1 diabetes subject and demonstrated their effect on transplantation efficiency. These results will lead to further understanding of the pathogenesis of type 1 diabetes. Regenerative therapy using stem cells that regenerate beta cells would be a promising approach to cure type 1 diabetes, in which insulin-producing pancreatic beta cells are damaged (Chhabra and Brayman, 2013). Therefore, our results may contribute to improvements in protocols for preparing ADSCs from patients with T1DM. In summary, the present study provided further characteristics of T1DM ADSCs that are used for regenerative therapy.

## Conclusion

In this study, we successfully identified *AMFR* and *DCTN2* as differentially expressed genes in immunodeficient mice transplanted with T1DM ADSCs. We also found that knocking down the gene expression of *AMFR* and *DCTN2* increased cell viability and stem cell ratio, and improved transplantation resistance in immunodeficient mice. This study was the first report, characterized stem cells from a patient with type 1 diabetes and demonstrated the efficacy of *AMFR* and *DCTN2* in stem cell transplantation. Suppression of *AMFR* and *DCTN2* expression is expected to enhance the efficacy of stem cell transplantation in patients with type 1 diabetes.

## Data Availability

The data presented in the study are deposited in the DNA Data Bank of Japan (DDBJ) repository, accession number PSUB018565.

## Abbreviations

T1DM, type 1 diabetes mellitus; T1DM ADSCs, T1DM adipose-derived stem cells; ADSCs, adipose-derived mesenchymal stem cells; ESCs, embryonic stem cells; iPSCs, induced pluripotent stem cells; BMSCs, bone marrow-derived mesenchymal stem cells; T2DM, type 2 diabetes mellitus; DAVID, the database for annotation, visualization, and integrated discovery; KEGG, the kyoto encyclopedia of genes and genomes; ER, endoplasmic reticulum; T2DM ADSCs, adipose-derived stem cells from type 2 diabetic patients.
