# Supplementary material for: AMFR and DCTN2 genes cause transplantation resistance of adipose-derived mesenchymal stem cells in type 1 diabetes mellitus
Source: Front Pharmacol. 2022 Oct 4;13:1005293. doi: 10.3389/fphar.2022.1005293 (PMC9577117; doi:10.3389/fphar.2022.1005293)
Supplement: Supplementary file 1 [file Table1.docx]

| **Reagent type (species)** | **Designation** | **Source** | **Identifiers** |
| --- | --- | --- | --- |
| Cell line (Homo sapiens) | Adipose-Derived Stem Cells Normal | Lonza | PT-5006 |
| Cell line (Homo sapiens) | Adipose-Derived Stem Cells T1DM | Lonza | PT-5007 |
| Culture medium | Adipose-Derived Stem Cells Growth Medium BulletKit™ | Lonza | PT-4505 |
| Reagent | Matrigel Basement Membrane Matrix For organoid formation Phenol red free | Corning | 356255 |
| Reagent | Matrigel Basement Membrane Matrix | Corning | 354234 |
| immunodeficient mice | BALB / cAJcl-*nu / nu* | CLEA Japan Inc. | nu / nu |
| Kit | RNeasy Mini Kit (250) | QIAGEN | 74136 |
| Reagent | NEBNext Poly(A) mRNA Magnetic Isolation Module | NEB | E7490 |
| Kit | NEBNext Ultra Ⅱ RNA Library Prep Kit for Illumina | NEB | E7770 |
| Software | CLC Genomics Workbench 12.0.3 | QIAGEN | 12.0.3 |
| Software | Database for Annotation, Visualization and Integrated Discovery (DAVID) | LHRI | 12734009 |
| Kit | PrimeScript™ RT reagent Kit (Perfect Real Time) | Takara Bio | RR037A |
| Reagent | SYBR® Premix Ex Taq™ II (Tli RNaseH Plus) | Takara Bio | RR820A |
| Reagent | Lipofectamine^TM^ LTX Reagent & Plus ™ Rreagent | Invitrogen | 15338100 |
| Kit | MTT Cell Proliferation and Cytotoxicity Assay Kit | BOSTER biological technology | AR1156 |
| Kit | Human Mesenchymal Stem Cell Verification Flow Kit | R&D systems | FMC020 |
| Software | FLOWJO v10 | BD Bioscience | 663335 |
| Software | Prism9 | GraphPad Inc. | academic ver. |
| Machine | Illumina NextSeq | Illumina | 550 |
| Machine | CO_2_ incubator DIRECT HEAT INCUBATOR astec | ASTEC CO.,LTD. | SCA-165D |
| Machine | StepOnePlus ™ Real-Time PCR System | applied biosystems | 4376592 |
| Machine | Flow cytometer SA3800 | Sony | SA3800 |

**Supplemental table 1. The list of materials**
