# Supplementary material for: AMFR and DCTN2 genes cause transplantation resistance of adipose-derived mesenchymal stem cells in type 1 diabetes mellitus
Source: Front Pharmacol. 2022 Oct 4;13:1005293. doi: 10.3389/fphar.2022.1005293 (PMC9577117; doi:10.3389/fphar.2022.1005293)
Supplement: Supplementary file 2 [file Table2.docx]

**Supplemental table 2. The list of qPCR primer.**

| ***Symbol*** | **Acc. No.** | **Forward** | **GC%** | **Tm** | **Reverse** | **GC%** | **Tm** |
| --- | --- | --- | --- | --- | --- | --- | --- |
| *ADCY3* | NM_001320613.2 | GCCCAACTTTGCTGACTTCTACAC | 50 | 63.5 | TGACTCCTGAAGCCGCCATA | 55 | 64.1 |
| *DCTN2* | NM_006400.4 | ATCAAGTGGAGGCTCGGCTAC | 57.1 | 62.9 | GACAAGTCTCTGCACCAGCTCA | 54.5 | 63.1 |
| *ARHGDI* | NM_001175.7 | GGATGACGATGATGAGCTGGAC | 54.5 | 64.2 | CGGGTGACAACGACATTGG | 57.9 | 63.4 |
| *ATXN3* | NR_028455.2 | AAATTCTGATCAGCTGACATCCTC | 41.7 | 61 | TGGTCACAGCTGCCTGAAG | 57.9 | 61.2 |
| *AMFR* | NM_001144.6 | TTTCCGAGCCTCCAGTCCA | 57.9 | 64.2 | AAACCAAAGCTTGCTGCCTAACC | 47.8 | 64.8 |
| *CANX* | NM_001363993.1 | GAGGCGAGTAATCCAGTTGGAGA | 52.2 | 64 | GCAGTTCCAAGCACCAGTAACA | 50 | 62.5 |
| *PDIA6* | NM_001282704.1 | TGCAGGCAGAAATTCTTATCTGG | 43.5 | 62.7 | ATCTTGCGTGCATTGATGG | 47.4 | 60.6 |
| *PRKCSH* | NM_002743.3 | CCCGCAACAAGTTCGAGGA | 57.9 | 64.6 | AGTTTGGGTTTCTGCGAGACAAG | 47.8 | 64 |
| *GAPDH* | NM_002046.4 | GCACCGTCAAGGCTGAGAAC | 60 | 63.3 | TGGTGAAGACGCCAGTGGA | 57.9 | 64 |
