# Supplementary material for: AMFR and DCTN2 genes cause transplantation resistance of adipose-derived mesenchymal stem cells in type 1 diabetes mellitus
Source: Front Pharmacol. 2022 Oct 4;13:1005293. doi: 10.3389/fphar.2022.1005293 (PMC9577117; doi:10.3389/fphar.2022.1005293)
Supplement: Supplementary file 3 [file Table3.docx]

**Supplemental table 3. The list of siRNA.**

| **Name** | **Sense/Antisense** | **Sequence** |
| --- | --- | --- |
| siRNA for *AMFR* | Sense | GCUCUGCAAGGAUCGAUUtt |
| siRNA for *AMFR* | Antisense | AAAUCGAUCCUUGCAGAGCtg |
| siRNA for *DCTN2* | Sense | GUAGAGCUGUUGCAAGCAAtt |
| siRNA for *DCTN2* | Antisense | UUGCUUGCAACAGCUCUACag |
